# Surfactant therapy for COVID-19 related ARDS: a retrospective case–control pilot study

**DOI:** 10.1186/s12931-020-01603-w

**Published:** 2021-01-18

**Authors:** Simone Piva, Robert M. DiBlasi, April E. Slee, Alan H. Jobe, Aldo M. Roccaro, Matteo Filippini, Nicola Latronico, Michele Bertoni, John C. Marshall, Michael A. Portman

**Affiliations:** 1 Department of Medical and Surgical Specialties, Radiological Sciences and Public Health, University of Brescia, Brescia, Italy; 2 Department of Anaesthesia, Critical Care and Emergency, Spedali Civili University Hospital, Piazzale Spedali Civili, 1, 25123 Brescia, Italy; 3 Respiratory Therapy Department, Seattle Children’s Hospital, Seattle, WA USA; 4 Center for Integrative Brain Research, Seattle Children’s Research Institute, Seattle, WA USA; 5 University College London, London, UK; 6 Perinatal Institute Cincinatti Children’s Hospital, Cincinnati, OH USA; 7 Children’s Hospital of Cincinnati, Cincinnati, OH USA; 8 University of Cincinatti, Cincinatti, OH USA; 9 Clinical Research Development and Phase I Unit ASST Spedali Civili Di Brescia, Brescia, Italy; 10 Li Ka Shing Knowledge Institute, Unity Health Toronto, University of Toronto, Toronto, ON Canada; 11 Department of Pediatrics, University of Washington School of Medicine, Seattle, WA USA

**Keywords:** Covid-19, Acute respiratory distress syndrome, Surfactant replacement therapy

## Abstract

**Background:**

COVID-19 causes acute respiratory distress syndrome (ARDS) and depletes the lungs of surfactant, leading to prolonged mechanical ventilation and death. The feasibility and safety of surfactant delivery in COVID-19 ARDS patients have not been established.

**Methods:**

We performed retrospective analyses of data from patients receiving off-label use of exogenous natural surfactant during the COVID-19 pandemic. Seven COVID-19 PCR positive ARDS patients received liquid Curosurf (720 mg) in 150 ml normal saline, divided into five 30 ml aliquots) and delivered via a bronchoscope into second-generation bronchi. Patients were matched with 14 comparable subjects receiving supportive care for ARDS during the same time period. Feasibility and safety were examined as well as the duration of mechanical ventilation and mortality.

**Results:**

Patients showed no evidence of acute decompensation following surfactant installation into minor bronchi. Cox regression showed a reduction of 28-days mortality within the surfactant group, though not significant. The surfactant did not increase the duration of ventilation, and health care providers did not convert to COVID-19 positive.

**Conclusions:**

Surfactant delivery through bronchoscopy at a dose of 720 mg in 150 ml normal saline is feasible and safe for COVID-19 ARDS patients and health care providers during the pandemic. Surfactant administration did not cause acute decompensation, may reduce mortality and mechanical ventilation duration in COVID-19 ARDS patients. This study supports the future performance of randomized clinical trials evaluating the efficacy of meticulous sub-bronchial lavage with surfactant as treatment for patients with COVID-19 ARDS.

## Background

The SARS-CoV-2 associated disease (COVID-19) has caused massive mortality worldwide. Those patients requiring hospitalization frequently have bilateral pneumonia, and about 15% develop ARDS [1]. Initially, COVID-19 produces diffuse alveolar damage, similar to the pathology seen in other forms of viral pneumonia, then followed by distinct angiocentric features: severe endothelial injury, microangiopathy, and occlusion of alveolar capillaries [2]. Clinically, this COVID-19 profile fulfils the ARDS definition but with distinctive features including severe hypoxemia associated with near-normal respiratory system compliance [3].

Thus, two different "phenotypes" have been identified in COVID-19 related ARDS. Early in the course (Type L: Low), patients display nearly normal compliance, low ventilation-to-perfusion (VA/Q) ratio, low lung weight, and low lung recruitability. Later (Type H: High), patients show low compliance, right-to-left shunting, high lung weight, and high lung recruitability [3]. The transition between the two phenotypes likely occurs from either progressively worsening viral pneumonia, or from ventilator-induced lung injury (VILI) [4]. Recent clinical experience suggests that type L patients should be managed by lower positive end-expiratory pressure (PEEP), slightly higher tidal volume (8–9 ml/kg), and prone position. Type H should be managed as severe ARDS, with higher PEEP, prone position, and extracorporeal support [4].

Pathogenic coronaviruses including SARS-CoV, MERS-CoV, and now SARS-CoV-2 use the ACE2 receptor to access, infect and destroy alveoli lining and surfactant producing type II pneumocytes [5]. Biopsy and post-mortem specimens in Covid-19 reveal diffuse alveolar damage, protein leak, inflammation in the alveolar walls, and desquamation of type II pneumocytes [6].

Notably, in ARDS, there is an impairment in lung surfactant activity and a reduction in the content and composition of active large surfactant aggregates, due to different mechanisms: the interaction between surfactant and inflammatory edema and the inhibition of surfactant aggregates formation [7]. With COVID-19 ARDS, surfactant depletion also may occur through virus-induced lysis of Type II pneumocytes with associated hyaline membrane formation [6], radiographic evidence of ground-glass opacities, and bilateral infiltrates, reduced pulmonary compliance, and refractory hypoxemia [8]. The pathophysiological findings in these critically ill adults are reminiscent of 'primary surfactant-deficiency' in preterm infants experiencing respiratory distress syndrome (RDS). Surfactant activity deficits can be mitigated by increasing the concentration of active surfactant [9]. Accordingly, the exogenous surfactant has proven effective in preterm infants with RDS when given appropriately [10].

Although surfactant therapy improves oxygenation and lung compliance in multiple animal models of ARDS [9–11], clinical trials failed to show any advantage of mortality in humans [12, 13]. The failure of these trials could be due to aspects of clinical trial design such as the timing of surfactant dosing [14], and method of administration. Early pilot studies in ARDS used targeted surfactant treatments with bronchoscopic guided installations [15]. A recent report shows that there is insufficient drug or volume in the dosing procedure for the surfactant to reach the alveoli in an adult lung because of the "Coating Cost" of the surface area of the airways [16]. There is a massive "fractal problem" to achieve a uniform distribution to the alveoli as with as many as 17 airway branch points to reach the 500 million alveoli in the adult lung [17].

Accordingly, a strong rationale exists for using surfactant in H type Covid-19 if these drug administration issues can be resolved. Therefore, we retrospectively analysed data from seven subjected to mechanical ventilation (MV) and treated with an approved 'off-label' Curosurf (Poractant Alfa, Chiesi, Italy), a porcine-derived surfactant, used worldwide for premature infant RDS.

Overall, we sought to test the hypothesis that a level of combined 'primary' and/or secondary VILI induced surfactant-deficiency occurs with COVID-19 ARDS, and the corollary that meticulous surfactant replacement can improve clinical outcome. Our first study objective was to test the feasibility and safety of surfactant instillation by bronchoscopy in SARS-CoV-2 patients. Secondary efficacy objectives were to determine: the effects of surfactant on (1) mechanical ventilation (MV) duration, and (2) on mortality.

## Methods

We conducted a single-center retrospective case–control study on prospectively collected data, on seven patients admitted to a single Intensive Care Unit (ICU) at Spedali Civili di Brescia University Hospital, affected by ARDS and with a positive qPCR for the SARS-CoV-2 virus (Covid-19), between March 3 and April 27, 2020. The study was conducted in accordance with the Declaration of Helsinki and Good Clinical Practice guidelines. This study was submitted to our local ethics committee (Comitato Etico di Brescia) as a retrospective analysis. Given the retrospective nature of this study and in accordance with current legislation, the need for informed consent was waived. We followed the STROBE (Strengthening the Reporting of Observational Studies in Epidemiology) guidelines for reporting results of this prospective cohort study [18]. The COVID-19 diagnosis was based on a positive qPCR for the SARS-CoV-2 virus. Patients were managed using World Health Organization's recommendations [19]. During the study period, 94 patients were admitted to our ICU; all matched the Berlin definition for severe ARDS.[20] Patients were selected to receive surfactant if they had a PaO_2_/FiO_2_ (P/F) ratio < 150, after all the other treatments, failed to improve oxygenation, and when extracorporeal membrane oxygenation could not be applied.

### Patients selection

Patient selection for off-label use of surfactant administration was based on careful selection of only H type patients, although its application was not always possible due to the limited resources. Once ARDS was diagnosed, patients were classified as Type L and Type H, based on compliance (cut-off level = 40 mL/cmH_2_O) [3]. Patients with suspected H Type Covid-19 were selected and divided into recruitable and non-recruitable based on Recruitment to Inflation Ratio (RIR) [21]. When recruitable (RIR > 0.5), patients underwent recruitment manoeuvres [22]; if patients had an RIR ≤ 0.5, a trial of inhaled Nitric Oxide (iNO) was given when available, following available protocol. In particular, a fixed dose of 20 ppm was administered, eventually increased to 40 ppm based on clinical response. iNO at 40 ppm was maintained for a maximum of 72 h, then reduced to 20 ppm until severe hypoxemia resolves. Stepwise iNO weaning was performed, decreasing iNO by 5 ppm every 4 h. Methaemoglobin was monitored during iNO administration [23, 24]. In both L and H Type, the prone position was applied when P/F ≤ 150 for at least 16 h with 4–8 h of the supine position [25]. When patients did not respond to any of the previous medical approaches, and extracorporeal circulation was not applicable (especially for the limited resources available), off-label Curosurf surfactant was administered. Patients' treatment with respect to iNO, prone position, recruiting manoeuvre and ECMO was not changed after surfactant application.

### Study procedure

Surfactant replacement was performed via bronchoscopy stepwise with time in between for recovery; no bronchoscopic secretion aspiration manoeuvres were performed before the study procedure. Curosurf (720 mg) was diluted in a total volume of 150 mL normal saline and subsequently divided into five separate aliquots of 30 ml (144 mg) [14, 16]. Patients were pre-oxygenated (F_IO2_ = 0.8–0.9) several minutes prior to the procedure. PEEP was increased by 10% to avoid lung de-recruitment. The surfactant was delivered via bronchoscopy in 15 ml aliquots administered in each 2nd generation bronchi. When possible and well-tolerated, the aliquots were divided into the 3rd generation bronchi. In order to avoid desaturation and de-recruitment, the bronchoscope was retracted following each instillation, and patients were allowed to ventilate for 1 min. At the end of the procedure, patients were placed back on pre-surfactant ventilator settings. Clinical staff was instructed to avoid tracheal aspiration after surfactant administration unless patients experienced desaturation or severe hypercarbia due to secretion accumulation and obstruction.

Safety was tested recording any adverse events, defined as any acute life-threatening deterioration of SpO_2_ or hemodynamics. Moreover, any acute change of lung compliance or driving pressure or increase in peak inspiratory pressures were recorded by the bedside team before, during, and after the procedure. Feasibility was evaluated by annotating any particular problem or stress experienced by ICU nurses or other clinicians when assisting in surfactant instillation manoeuvres.

Arterial blood gas analysis (ABG) was performed and recorded after 30 min and two hours following surfactant administration. ABG data were acquired from the patient medical record on the day prior to and 24 h following surfactant to assess response. Beyond that timeframe, the presence of ABG data was inconsistent, making longer-term assessment and comparison of P/F in treated and control subjects difficult. All procedures and peri-dosing monitoring were performed by the same operator (SP).

Feasibility objectives of the present study were: (1) the ability of the ICU team to recruit patients in a environment with very limited resources availability during the SARS-COV-2 pandemic, measured by the ability of the staff to perform the study procedure when clinicians identified patients that potentially matched the criteria for study enrollment; (2) the ability of the operator (SP) to perform the bronchoscopy procedure in ARDS COVID-19 patients and (3) the ability to the collect and record patient' data with Research Electronic Data Capture (REDCap), a secure web application for building and managing online surveys and databases. Safety was tested by tracking any severe adverse event judged as being directly correlated to the protocol application—any major cardiac events (cardiac arrest, hemodynamically significant arrhythmias, acute coronary syndrome), or respiratory event (respiratory arrest)—was recorded. Moreover, the operator (SP) underwent a serological test for COVID-19 on April 20.

### Data management and statistical analysis

The RedCap database was used to record data, including patients' demographics and past medical history. On a daily basis, data on ventilator parameters, blood-works, clinical parameters were also recorded for the entire population. Duration of mechanical ventilation (MV), reintubation rate, ICU outcome (discharge to the ward, death or other step-down ICU), and hospital outcome (alive, dead, or still in hospital) were evaluated at least 28 days after treatment. Continuous variables are presented as mean (standard deviation, SD) or median (interquartile range, IQR); qualitative variables are summarized as counts and percentages.

Controls were matched to surfactant cases in a 2:1 ratio by whether a tracheostomy had been performed, P/F was within 30, duration since the first day of MV (within one day), and body mass index (within 5 kg/m^2^). Controls were treated within the same time period as cases. Operationally, a greedy matching algorithm developed at Mayo Clinic was used to conduct this matching [26]. For each control patient, values for each ICU day (constant BMI, time-dependent tracheostomy, P/F ratio, and days since the start of mechanical ventilation) were available for matching. Baseline time was defined for controls as the day prior to surfactant administration for the matched treated patients. Mechanical ventilation baseline characteristics (values of SpO_2_, PEEP, Pplat, driving pressure, pH, pCO_2_, Lactate, and bicarbonate, on the day of surfactant administration or on a matching day) were compared using conditional logistic regression (modelling the probability of status as a case versus control) to adjust for matching variables. Outcomes were analysed using generalized linear models for time-dependent continuous variables (P/F ratio) controlling for matching variables and adjusted for repeated measurements within subjects. Time-dependent outcomes were assessed using Cox proportional hazards models controlling for matching variables. For survival and duration of MV, the time variable was defined as the time in days from baseline until discontinuation of MV or death. The analysis for the duration of MV was based on time to first extubation or removal from a ventilator for tracheostomized patients, censored at last known date of MV for patients who were transferred to another ICU while ventilated. Patients were censored for survival at the last known date when the patient was alive.

## Results

Seven patients with H type Covid-19 were treated with surfactant during the study period, for a total of 10 surfactant instillations. Overall, five patients were administered a single (750 mg) surfactant dose, and two patients received two separate doses (750 mg) administered over multiple days. In Table 1, case and control's demographic characteristics are represented. There were no differences in terms of age, gender, body mass index (BMI), ICU admission SAPS II, and comorbidity between case and control. Baseline mechanical ventilation characteristics did not differ between case and control (Table 2). Duration of MV before matching, ICU-LOS before matching, insertion of tracheostomy, BMI, and P/F ratio are shown, for both case and control, in Additional file 1: Table S1. Patient treatment before matching, including neuromuscular blocking agents, iNO, Pronation, Tocilizumab administration, Steroids administration, antiviral, and Chlorochine/Hydroxychloroquine administration, were not different in case and control (Additional file 1: Table S2).

**Table 1:** Demographics characteristics of case and control

	Surfactant (n = 7, 0.33%)	Control (n = 14, 0.66%)	P value
Age, mean (SD)	66.14 (4.33)	60.85 (10.79)	0.232
Gender male, n (%)	6 (85.7%)	11(78.6%)	0.873
BMI, mean (SD)	28.60 (4.54)	28.71 (4.49)	0.958
SAPSII, mean (SD)	49.14 (12.30)	43.92 (15.42)	0.447
Cardiopathy, n (%)	5 (71.4%)	4 (28.5%)	0.124
Diabetes, n (%)	1 (14.3%)	2 (14.3%)	0.247
Immunodepression, n (%)	0 (0%)	2 (14.3%)	0.530
Obesity, n (%)	2 (28.6%)	3 (21.4%)	0.998
Pneumopathy, n (%)	1 (14.3%)	0 (0%)	0.335

*BMI* body mass index (weight in kilograms divided by the square of the height in meters)

**Table 2:** Conditional logistic regression for baseline mechanical ventilation characteristics

	Surfactant (N = 7)	Control (N = 14)	P-value
S_pO2_ (worst value), %	92.9 ± 3.80	94.8 ± 3.27	0.161
Mean P/F ratio (SD)	134.14 ± 12.84	137.00 ± 8.33	0.850
Mean (SD) PEEP, mmHg	9.14 ± 3.02	11.7 ± 3.05	0.113
Mean (SD) Pplat, mmHg	23.2 ± 1.72	22.5 ± 2.81	0.674
Mean (SD) Driving pressure	14.5 ± 2.74	10.2 ± 3.49	0.467
Mean (SD) pH	7.34 ± 0.11	7.41 ± 0.08	0.083
Mean Log Pa_CO2,_ mmHg	4.06 ± 0.25	3.91 ± 0.26	0.109
Mean (SD) Lactate, mmol/L	1.23 ± 0.38	1.46 ± 0.63	0.364
Mean (SD) Log Bicarbonate, mEq/L	3.44 ± 0.18	3.40 ± 0.13	0.429

*BMI* body mass index, *Pplat* plateau pressure

P/F ratio showed no statistical difference at 2 h following surfactant administration compared to baseline [mean change (SD) 13.2% (72)]. The pH, PaCO_2,_ and static lung compliance did not change over the pre/post-dosing period on the day of treatment, suggesting stable lung function following surfactant administration. There was no significant difference in P/F ratio between case and control groups over the days following surfactant administration. However, a trend in improvement in P/F ratio over time was more pronounced in the surfactant group than in the control group (Additional file 1: Table S3 and Additional file 1: Figure S1).

Outcomes measures and comparisons between treated and control patients are shown in Table 3. Six (85.7%) cases and 5 (35.7%) controls were transferred from ICU and weaned from mechanical ventilation, p = 0.06. One (14.3%) of the case and 6 (42.9%) control were reintubated in ICU after extubation failure, p = 0.337.

**Table 3:** Outcomes

	Duration of MV*	Transferred ventilated***	ICU outcome	Hospital outcome	Days outcome evaluation
Case 1	13	No	Ward	Alive	55
Control 1–1	11	Yes	Dead	Dead	65
Control 1–2	36	Yes	ICU	Alive	52
Case 2	22	Yes	Dead	Dead	55
Control 2–1	19	No	Ward	Alive	44
Control 2–2	20	Yes	Dead	Dead	42
Case 3	27	No	ICU	Alive^†^	49
Control 3–1	24	No	Ward	Alive	50
Control 3–2	21	Yes	Ward	Alive^‡^	47
Case 4	7	No	ICU	Alive	45
Control 4–1	16	Yes	Dead	Dead	71
Control 4–2	10	No	Ward	Alive	57
Case 5	16	No	Ward	Alive	37
Control 5–1	16	Yes	Ward	Dead	74
Control 5–2	16	Yes	Ward	Dead	41
Case 6	25	No	ICU	Alive	34
Control 6–1	28	No	ICU	Alive	51
Control 6–2	12	No	Ward	Alive	50
Case 7	10	No	Ward	Alive	32
Control 7–1	6	Yes	Dead	Dead	71
Control 7–2	9	Yes	Dead	Dead	48

*Duration of mechanical ventilation (MV), **ICU-Los = total days in ICU, ***Yes = patients transferred ventilated or dead in ICU

^†^Alive still In ICU, ^‡^patients alive still in hospital

ICU mortality rate was 1 (14.3%) in cases, and 5 (35.7%) in controls, p = 0.613. The 28-day mortality rate was 1 (14.3%) in cases and 9 (64%) in control, p = 0.063. Cox-proportional adjusted analysis on survival (evaluated at least at 28 days) yielded an HR of 0.12 (95% C.I. 0.01–1.36) of death in patients receiving surfactant, though not significant. Patients receiving surfactant had a shorter duration of MV with a hazard ratio (HR) of 2.42 (95% CI: 0.54–10.95), although not significant.

None of the patients experienced adverse events or desaturation during the surfactant administration, or a decrease in PaO_2_ or an increase in PaCO_2_ within 2 h following instillation. Clinical staff expressed that the procedure was safe and 'feasible' in all cases.

## Discussion

We provide one of the first reports on surfactant administration for COVID-19 H type ARDS. Surfactant was administered according to the off-label use guidelines, and primarily as rescue therapy for severe ARDS. Accordingly, we could assess feasibility and safety but only provide preliminary and limited data regarding drug effect. No patient receiving surfactant exhibited acute decompensation. Despite concerns of risks to healthcare providers by bronchoscopy procedures, the personnel involved in these procedures did not contract COVID-19. Thus, our data suggest that exogenous surfactant installation via bronchoscopy represents a safe and feasible option in patients with severe (H type) COVID-19 ARDS. Considering the lack of a prospective randomized design, we used a very strict matching algorithm to compare treated patients to those untreated with surfactant but with similar disease severity. Eligible patients were randomly chosen for drug administration based on the availability of appropriate resources, including health care providers and support staff during the peak COVID-19 pandemic. Surfactant delivery in our study was performed in a single-center, following a uniform protocol with meticulous delivery and retention in the lungs. These preliminary data suggest that our surfactant delivery strategy if performed within a randomized control design and adequately powered, could prove to reduce overall time on mechanical ventilation and long term (28 days) mortality.

Treatment approaches for COVID-19 ARDS are rapidly emerging, but mortality exceeding associated with VILI is reported to be 60–85% in some case series [27]. Even survivors require inordinately long periods of MV and sustain accompanying comorbidities. As such, any reduction in MV duration has the potential to reduce VILI and mortality. Liquid surfactant instilled via a catheter through endotracheal tubes reduces ventilation time in premature infants but can cause peri-dosing complications, including agitation/discomfort, refractory hypoxemia, bradycardia, hemodynamic instability, and airway occlusion [10]. Unlike prior clinical trials in RDS or ARDS [28], we did not observe any of these immediate severe adverse events. A recent meta-analysis of 11 randomized clinical trials in over 3000 patients showed how surfactant administration did not improve mortality or disease severity (P/F ratio) in adult patients with ARDS [28]. However, these studies were all negatively impacted by extreme patient heterogeneity and high prevalence of patients with septic shock. Some studies were terminated as they showed substantial numbers of adverse events in the treatment groups: liquid instillation was poorly tolerated, airway obstruction and increased hypoxia [25]. Other studies were terminated due to futility [29, 30].

Dosing and surfactant formulation are also major considerations and possible sources of failure in prior studies. Findings from adult studies have reported some improvement in oxygenation using high surfactant doses from 250 to 300 mg/kg [28], that necessitates an instilled surfactant volume of 280–400 ml, which in most cases requires a total volume that could exceed the pre-set tidal volume [27]. Instillation of liquid surfactant at these doses and volumes could potentially overwhelm the cardiopulmonary system and add to respiratory failure and deterioration. Moreover, transient changes in regional lung mechanics following drug administration could affect the distribution of tidal volume and increase the risk for VILI in patients who may already be compromised and not able to tolerate these large fluid volumes [28].

The method by which surfactant was instilled into the lungs suggests that inadequate alveolar delivery may be a major cause for the failure of these later studies [16, 29]. In theory, bypassing the upper airways with a bronchoscope and administering a drug to segmental and lobar bronchi could substantially reduce the drug volume, as well as prevent drug loss to large airways to improve medication delivery to lobar airspaces where it is needed most. This is particularly relevant for Covid-19 patients who may have non-uniform or 'heterogenous' lung disease.

Prior studies have evaluated surfactant delivery via bronchoscope for distribution to individual lobes for ARDS. Walmrath et al. [31] used bovine surfactant extract (300 mg/kg Alveofact; 23 g diluted in ~ 400 mL) delivered in divided doses to each segment of the lungs via flexible bronchoscope to ARDs patients with septic shock. Similar to our findings, they showed surfactant to be feasible and safe in terms of gas exchange, lung mechanics, and hemodynamics. Despite observing a nearly two-fold increase in P/F in subjects, they reported a 44% mortality in treated subjects. Gunther et al. [30] used multiple doses of natural bovine surfactant (300–500 mg/kg) via bronchoscope and showed improved biophysical surfactant activity and similar P/F but with higher mortality (40%) than those patients treated with surfactant in the current study (15%). When administering 50 mg/kg Curosurf in severe ARDS, Spragg et al. [14] used a similar bronchoscopic volume and delivery strategy as the current study. However, all aliquots were suctioned from the lungs shortly following instillation. Patients treated with this 'surfactant lavage procedure' did not have adverse effects but showed only limited improvement in gas exchange compared to controls. Similar to Spragg et al. [14], diluted surfactant in this study was used as a 'lavage' and gently suctioned following 5 s to prevent surfactant from being inactivated by inflammatory mediators. This strategy lowered pulmonary inflammation scores in ARDS patients (n = 5) more than leaving liquid surfactant to absorb in the lungs. That study did not report length of MV or mortality in the subjects or compare outcomes between surfactant cases and controls.

We left the surfactant in the lung for a 4-h period and requested that clinical staff avoid endotracheal suctioning. We did not observe major changes in lung mechanics or P/F, which are often attributed to surfactant volume retention. This strategy also did not produce any adverse effects. Meticulous surfactant delivery into second and third generation bronchi was not proscribed in previously published study methods, nor was surfactant retention in airways after the bolus. In contrast, surfactant delivery in our study was performed by a single individual, following a uniform protocol with accurate delivery and retention of surfactant in the bronchi, and using near recommended dosing per kg for a natural surfactant. Disease heterogeneity was also minimized as these patients all had SARS-COV-2 induced severe ARDS.

Most patients from our study only received a single surfactant dose. While adult ARDS trials were high-profile failures, investigation of fluid dynamic modelling and review of the original studies that showed efficacy suggest that larger fluid volumes with high concentration surfactant and distal intrabronchial drug delivery are advantageous and maximize alveolar surfactant delivery [16]. The influence of gravity and on the liquid surfactant plug splitting at airway bifurcations is far greater in the adult than in premature infants due to greater patient size, which may impact the homogeneity of delivery. Based on the prior studies [33], we used diluted surfactant with proportionally higher saline volumes than typically used in infants. This approach reduced drug viscosity and avoided the requisite "coating cost" of the drug in the airways. For a 70 kg adult, our dilution yielded a surfactant concentration of ~ 10 mg/kg with a total volume of ~ 2.1 mL/kg per treatment. This approximates the weight-based volume (mL/kg) but is a fraction of the concentration used in preterm infants (2.5 mL/kg = 200 mg/kg). Unlike previous high concentration/high volume approaches with ARDS, this dilution may be a more efficient alternative for optimization. Lobar administration via bronchoscopy does require close monitoring, longer administration time, and the services of an experienced bronchoscopist.

We worked under the limitations of a retrospective analysis. This includes some data loss, particularly when patients were transferred from the ICU. As an off-label use of the investigated drug, we could enroll a low number of subjects, thus impacting the power for assessing the efficacy of our protocol. Finally, the selection of patients was influenced by the availability of resources, including the bronchoscope during the pandemic.

## Conclusions

These data support the hypothesis that some level of combined 'primary' and/or secondary VILI induced surfactant-deficiency occurs with COVID-19 ARDS. Thus, a rationale exists for pursuing strategies that will reduce or prevent endotracheal intubation and duration of mechanical ventilation in patients with COVID-19 [4]. These strategies could include bronchoscopic and other forms of surfactant delivery. The current data show that liquid surfactant delivery to patients with SARS-COV-19 induced ARDS is feasible and well-tolerated. Meticulous bronchoscopic delivery avoids acute decompensation. Surfactant efficacy in reducing mechanical ventilation time still needs to be established by careful randomized clinical trials.

## Supplementary Information

Additional file 1: **Table S1**: Representation of matching variables for each case and control.** Table S2**: Comparison of therapeutic strategies between surfactant Group and Control Group before matching. **Table S3: **Comparison of P/F ratio between Surfactant group and Control Group after matching. **Figure S1: **Comparison of P/F ratio between Surfactant group and Control Group after matching.

## Data Availability

The datasets analysed during the current study are available in the GitHub repository, available at https://github.com/pivadoc/Surfactant/issues/1.

## Abbreviations

COVID-19: SARS-CoV-2 associated disease
ARDS: Acute respiratory distress syndrome
VA/Q: Ventilation-to perfusion
VILI: Ventilator-induced lung injury
PEEP: End-expiratory pressure
RDS: Respiratory distress syndrome
MV: Mechanical ventilation
RIR: Recruitment to inflation ratio
ABG: Arterial blood gas analysis
P/F: PaO_2_/FiO_2_
SD: Standard deviation
IQR: Interquartile range
ICU: Intensive care unit
iNO: Inhaled nitric oxide
