# Supplementary material for: Surfactant therapy for COVID-19 related ARDS: a retrospective case–control pilot study
Source: Respir Res. 2021 Jan 18;22:20. doi: 10.1186/s12931-020-01603-w (PMC7812332; doi:10.1186/s12931-020-01603-w)
Supplement: Supplementary file 1 — Additional file 1: Table S1: Representation of matching variables for each case and control. Table S2: Comparison of therapeutic strategies between surfactant Group and Control Group before matching. Table S3: Comparison of P/F ratio between Surfactant group and Control Group after matching. Figure S1: Comparison of P/F ratio between Surfactant group and Control Group after matching. [file 12931_2020_1603_MOESM1_ESM.docx]

**Supplementary Materials Table 1**: Representation of matching variables for each case and control.

|  | MV * | ICU-LOS** | Tracheostomy | P/F |
| --- | --- | --- | --- | --- |
| CASE 1 | 2 | 3 | Yes | 142 |
| CONTROL 1-1 | 2 | 2 | Yes | 153 |
| CONTROL 1-2 | 2 | 2 | Yes | 164 |
| CASE 2 | 7 | 7 | Yes | 84 |
| CONTROL 2-1 | 6 | 6 | Yes | 91 |
| CONTROL 2-2 | 6 | 6 | Yes | 90 |
| CASE 3 | 4 | 4 | Yes | 118 |
| CONTROL 3-1 | 4 | 4 | Yes | 121 |
| CONTROL 3-2 | 5 | 5 | Yes | 120 |
| CASE 4 | 1 | 1 | No | 145 |
| CONTROL 4-1 | 1 | 1 | No | 145 |
| CONTROL 4-2 | 1 | 1 | No | 142 |
| CASE 5 | 4 | 5 | Yes | 163 |
| CONTROL 5-1 | 4 | 4 | Yes | 164 |
| CONTROL 5-2 | 3 | 3 | Yes | 165 |
| CASE 6 | 3 | 3 | Yes | 105 |
| CONTROL 6-1 | 2 | 2 | Yes | 106 |
| CONTROL 6-2 | 2 | 2 | Yes | 104 |
| CASE 7 | 1 | 1 | No | 182 |
| CONTROL 7-1 | 1 | 2 | No | 178 |
| CONTROL 7-2 | 2 | 3 | No | 175 |

* ICU LOS before matching. **MV duration before matching . *** Log of the value

Abbreviation: DP. Driving Pressure; PPlat. Plateau Pressure; NMBAs. neuromuscular blocking agents ; NO. Nitric Oxide

**Supplementary Table 2**: Comparison of therapeutic strategies between surfactant Group and Control Group before matching.

|  | | **Surfactant** | **Control** | **P-value** |
| --- | --- | --- | --- | --- |
| Neuromuscular blocking agents (NMBA). | Yes | 7 (100.0%) | 17 (81.0%) | 0.9762 |
|  | No | 0 (0.0%) | 4 (19.0%) |  |
| Nitric Oxide | Yes | 1 (14.3%) | 2 (9.5%) | 0.7209 |
|  | No | 6 (85.7%) | 19 (90.5%) |  |
| Pronation | Yes | 5 (71.4%) | 9 (42.9%) | 0.1164 |
|  | No | 2 (28.6%) | 12 (57.1%) |  |
| Tocilizumab | Yes | 1 (14.3%) | 6 (28.6%) | 0.5208 |
|  | No | 6 (85.7%) | 15 (71.4%) |  |
| Steroids | Yes | 2 (28.6%) | 15 (71.4%) | 0.0613 |
|  | No | 5 (71.4%) | 6 (28.6%) |  |
| Antiviral (Darunavir/Ritonavir/Lopinavir) | Yes | 4 (57.1%) | 15 (71.4%) | 0.4052 |
|  | No | 3 (42.9%) | 6 (28.6%) |  |
| Chloroquine/Hydroxychloroquine | Yes | 7 (100.0%) | 19 (90.5%) | 0.9760 |
|  | No | 0 (0.0%) | 2 (9.5%) |  |

**Supplementary materials Table 3:** Comparison of P/F ratio between Surfactant group and Control Group after matching.

|  | Surfactant | | Control | |  |
| --- | --- | --- | --- | --- | --- |
| Day* | Mean P/F | SD | Mean P/F | SD | p |
| 0 | 134.14 | 12.84 | 137.00 | 8.33 | 0.850 |
| 1 | 116.29 | 13.35 | 163.57 | 14.49 | 0.151 |
| 2 | 144.57 | 15.03 | 161.35 | 16.22 | 0.517 |
| 3 | 165.28 | 24.15 | 146.35 | 13.52 | 0.468 |
| 4 | 150.14 | 19.821 | 170.50 | 17.85 | 0.491 |
| 5 | 148.14 | 19.31 | 171.30 | 17.54 | 0.417 |
| 6 | 130.85 | 24.11 | 175.50 | 12.05 | 0.158 |
| 7 | 161.29 | 46.68 | 175.08 | 24.35 | 0.661 |
| 8 | 192.71 | 43.1 | 191.66 | 28.29 | 0.976 |
| 9 | 175.28 | 30.05 | 186.00 | 36.29 | 0.662 |
| 10 | 208.57 | 37.01 | 170.30 | 34.347 | 0.442 |
| 11 | 214.14 | 35.09 | 180.44 | 38.64 | 0.508 |

* Day: The number is referred to the day from matching (for surfactant group the day of surfactant administration; i.e.: day 2 is 24 hrs after administration.

Abbreviation: P/F: PaO_2_/FiO_2_ Ratio; SD: Standard deviation.

**Supplementary Material Figure 1:** Comparison of P/F ratio between Surfactant group and Control Group after matching.
